# 516 Fluid Resuscitation and Acute Kidney Injury in the Electric Burn Patient. Reconsidering Initial Resuscitation Goals

**DOI:** 10.1093/jbcr/irae036.151

**Published:** 2024-04-17

**Authors:** Sandra J Bernal, Laura S Nasiff, Norberto Navarrete

**Affiliations:** Universidad del Rosario, BOGOTÁ, Distrito Capital de Bogota; Universidad del Rosario, BOGOTÁ, Distrito Capital de Bogota; Hospital Simón Bolívar ESE, CAJICÁ, Cundinamarca; Universidad del Rosario, BOGOTÁ, Distrito Capital de Bogota; Universidad del Rosario, BOGOTÁ, Distrito Capital de Bogota; Hospital Simón Bolívar ESE, CAJICÁ, Cundinamarca; Universidad del Rosario, BOGOTÁ, Distrito Capital de Bogota; Universidad del Rosario, BOGOTÁ, Distrito Capital de Bogota; Hospital Simón Bolívar ESE, CAJICÁ, Cundinamarca

## Abstract

**Introduction:**

In burn patients, multiple studies have confirmed the risks of acute kidney injury (AKI) and mortality attributed to both under or over-volume resuscitation. Nonetheless, electrical burns patients are systematically excluded from these studies due to their unique pathophysiology, high incidence of rhabdomyolysis, and poor correlation between TBSA and injury severity. Recommendations regarding volume management vary and remain controversial as the literature is limited. The objective of this study is to describe fluid resuscitation while concurrently evaluating its association between sub-optimal or excessive fluid administration and the incidence of early AKI (eAKI).

**Methods:**

Retrospective cohort study. Registry data for adult patients admitted to Burn ICU during the first 48 hours after electrical injury, between 2007 to 2013 was analyzed. Descriptive statistics were calculated. The primary outcome was eAKI, according to the AKI network criteria. eAKI was modeled using multivariable logistic regression, including age, sex, TBSA, time to treat (between injury to start of treatment), and creatine phosphokinase maximum (CPKmax). CPK, fluid requirements, and urine output (UO) were recorded in the first week (Day 0-6 of admission). The protocol targets were UO >1.5 ml/kg/h in rhabdomyolysis or >2 ml/kg/h if macroscopic myoglobinuria were present.

**Results:**

A total of 456 patients were included. The median TBSA was 5% (2%-12%). 27 patients (5,9%) developed eAKI. None of the patients underwent renal replacement therapy. There were no significant differences in age, sex, CPK, or CPKmax value between eAKI and Non-eAKI patients, except for CPK3 (p=0.0483). Although volume infused in 0-2 days were lower in the eAKI group, there were no significant differences regarding under or over-resuscitation frequency and the hourly fluid resuscitation volume. The UO0 and UO1 (ml/hr) were significantly lower in the eAKI group (37.9 v. 139.1 ml/hr, p< 0.001 and 162.0 v. 187.6 ml/hr, p=0.005 respectively). The UO0 was the only variable independently associated with the development of eAKI (OR: 0.984, 95% CI: 0.976 - 0.992).

**Conclusions:**

Neither the time to treat nor the fluid volume resuscitation are associated with the development of eAKI in electrical injuries. However, reduced diuresis during the day of admission is associated with the development of eAKI.

**Applicability of Research to Practice:**

Estimating fluid volume according to TBSA is insufficient in electrical injuries, mainly when TBSA is less than 12%. Crystalloid infusion guided by urinary output may result in a lower incidence of eAKI.

